# Comparison between direct contact and extract exposure methods for PFO cytotoxicity evaluation

**DOI:** 10.1038/s41598-018-19428-5

**Published:** 2018-01-23

**Authors:** Girish K. Srivastava, Maria L. Alonso-Alonso, Ivan Fernandez-Bueno, Maria T. Garcia-Gutierrez, Fernando Rull, Jesús Medina, Rosa M. Coco, J. Carlos Pastor

**Affiliations:** 1 Instituto Universitario de Oftalmobiologia Aplicada (IOBA), Universidad de Valladolid, Valladolid, Spain; 2 Centro en Red de Medicina Regenerativa y Terapia Celular, Valladolid, Spain; 3 Red Temática de Investigación Cooperativa en Salud (RETICS), Oftared, Instituto de Salud Carlos III, Madrid, Spain; 4 Vision I+D, Valladolid, Spain; 5 Departamento de Física de la Materia Condensada, Cristalografía y Mineralogía, Universidad de Valladolid, Valladolid, Spain; 6 Hospital Clínico Universitario de Valladolid, Valladolid, Spain

## Abstract

A series of recent acute blindness cases following non–complicated retinal detachment surgery caused the release of several health alerts in Spain. The blindness was attributed to certain lots of perfluoro-octane (PFO; a volatile and transient medical device). Similar cases have been reported in other countries. This has raised questions regarding the validity of cytotoxicity test methods currently used to certify the safety of PFO lots. The tests were performed according to the International Organization for Standardization (ISO) norms, using the extract dilution method or the indirect contact method as applied to L929 cells, a line derived from mouse fibroblasts. The limitations of those methods have been resolved in this study by proposing a new cytotoxicity test method for volatile substances. The new method requires direct contact of the tested substance with cells that are similar to those exposed to the substance in the clinical setting. This approach includes a few new technical steps that are crucial for detecting cytotoxicity. Our new method detected toxic PFO lots that corresponded to the lots producing clinical blindness, which previous methods failed to detect. The study suggests applying this new method to avoid occurrence of such cases of blindness.

## Introduction

Testing the safety of a medical device and establishing the proper biocompatible mechanical, physical, and chemical properties of the device are critical for the safe use in clinical practice (Council Directive 93/42/EEC of 14 June 1993 Concerning Medical Devices). Using the correct test method to probe the safety is an important concern for manufacturing companies and certification authorities. The International Organization for Standardization (ISO) has released a series of norms (regulatory standards ISO 1993) and recommendations to be applied rigorously for certifying the safety of a medical device for clinical use. It recommends methods that include, though not limited to, analysing cytotoxicity, sensitization, intradermal irritation, and acute systemic toxicity.

Perfluoro-n-octane (PFO), also known as octadecafluorooctane, is a perfluorocarbon liquid that is synthesized as a perfluorinated derivative of the hydrocarbon octane. The molecular formula is C_8_F_18_, with a molar mass of 438.06 g/mol, melting point of −13 °F (−25 °C), and density of 1.77 g/cm³. It is one of the most popular and widely accepted intraoperative mechanical tools (medical devices) applied as a retinal manipulator for vitroretinal surgery in patients with retinal detachment (RD)^1^ as well as for floating lens fragments and luxated intraocular lenses^2,3^. Manufacturers have developed various techniques to synthesize and purify PFOs. One of them is electrochemical fluorination of octane to produce the raw PFO, followed by distillation and filtration for purification. PFO samples are tested for safety, and then packed in syringes or bottles of 5–7 ml that are supplied commercially^4^.

In recent years, various national health authorities have released notifications for cases of blindness following vitreoretinal surgery. Some of these notifications originating from the health authorities are: notification Ref. PS, 19/2015 of the Agencia Española de Medicamentos y Productos Sanitarios; the FSCA reference 091215-749 of the Swiss Agency for Therapeutic Products; notification 27082013 of the Agencia Nacional de Medicamentos of Chile; and notification 17/12/2015 of the Therapeutic Goods Administration (TGA) of Australia. These notifications confirm the failures of test methods used to measure the safety of PFO samples. In 2014–2015, several retinologists in Spain reported many cases of serious complications after apparent successful pars plana vitrectomy^5^. All of these cases used PFO (Ala-Octa®) of a German company (Alamedics, Dornstadt, Germany). The PFO had been tested by Eurofins, Planneg/Munich, Germany, a BioPharma Product testing company, using a test method that followed the ISO 10993-1; ISO 10993-5, and ISO 10993-12 norms.

Cytotoxicity testing is a primary method for establishing the safety of a medical device^6^. This pilot test drives the destiny of a medical device towards further testing, modification, or abandonment at the initial stages of development^7^. Current advancements in science and technology provide several standard cytotoxicity test methods that are sufficiently sensitive to detect various levels of cellular toxicity, i.e., from low to high. These tests can rapidly produce results that are suitable for quantitative assessment^6,7^. Many of them are described in the ISO guidelines (10993-12:2007 Part 12 Preparation of Samples and Reference Materials, UNE-EN ISO 10993-5:2009 Part 5 *In Vitro* Cytotoxicity Test, Part 5 section C.2.3.3 Verification of the Quality of Analysis (II); blank).

Selecting the best method for cytotoxicity evaluation of a medical device depends on the manufacturer or the company selling the product and the certification authorities. The ISO 10993-5 guideline recommends three types of cytotoxicity test methods: extract dilution, direct contact, and indirect contact test^6^. The extract dilution exposure method is applied to a wide variety of medical devices to detect toxins leached from exposed surfaces. The direct contact method is highly sensitive and able to detect weak cytotoxicity. The method applied by the German BioPharma Product Testing company for the toxic PFOs was the extract dilution exposure method. They tested the PFO with cultures of the mouse fibroblast L929 cell line. However this method, although valid from the ISO norms point of view, is far removed from clinical usage where PFO is injected into the ocular vitreous cavity where it is in direct contact with the neuroretina.

This paper describes a new protocol based on the direct cytotoxicity test method and includes new technical steps that are crucial for testing highly volatile medical devices such as PFO. Further, we validated the results by comparing the outcomes with data obtained by the extract dilution exposure method that is used to certify such devices safe for use. Both methods were performed following the EU-ISO norms. Although the European Norm (EN) is the same as the International Norm (ISO), here we argue that, at least in the European Union (EU), the established test methods for evaluating the toxicity of medical devices containing volatile substances are inadequate and must be changed to prevent new toxicological problems in the future.

## Methods and Materials

### Perfluoro-n-octane (PFO)

This study included different presumed toxic lots of PFO (Ala-Octa®) provided by the Basque Country Health Authorities, Innova Ocular, and, at the request of the Spanish Health Authorities, the Spanish dealer of the German manufacturer company. The company itself provided two presumed controls, one packaged in 2013 and the other in 2015. The lots were labelled by the company according to the packaging date (DD/MM/YY). The raw materials were obtained from a Russian supplier (trade name unknown to us) in 20-liter tanks from two different manufacturing lots: 1205052 and 1225572. The German company, Alamedics, periodically extracted 2-liter volumes and re-packaged them in 5-ml or 7-ml vials identified as “lots”. Table 1 provides a list of different lots from this company that were analysed for this study. As controls for this study, PFO samples that were in clinical use were supplied by two different manufacturers and commercial companies distributing the PFO lots (dealers).

**Table 1:** Summary of PFO lot sources and comparison of results obtained by the direct contact and extract dilution exposure methods.

Containers (PFO lots according to manufacturer)		PFO lots (according to manufacturer assigned DD/MM/YY)	Cytotoxicity assay (% cell culture viability)					
			Direct contact method with new technical steps					Extract contact method
			ARPE-19 cell cultures				L929 cell cultures	L929 cell cultures
			30 minutes*		60 minutes*		30 minutes*	72 hours***
			24 hours**	72 hours**	24 hours**	72 hours**	24 hours**	
1225572	Container 1	180214	59%	89%	25%	29%	NT	>70%
		150414	34%	NT	NT	NT	NT	>70%
		050514	50%	NT	NT	NT	32%	NT
	Mixed container	080714	53%	90%	30%	50%	NT	>70%
	Container 2	061014	1%	NT	NT	NT	1%	>70%
		171214	1%	NT	NT	NT	NT	>70%
Unknown container		210715	95%	NT	NT	NT	100%	>70%
Control 1		Non-Alamedics PFO manufacturer	>70%	>70%	>70%	>70%	>70%	>70%
Control 2		Stirred cell culture medium	NT	NT	NT	NT	NT	>70%
Control 3		Fresh Cell culture medium	100%	100%	100%	100%	100%	100%

*Cell culture contact period with PFO samples; **Cell culture growth period after PFO contact; ***Cell culture incubation with PFO extract; NT = Not tested or not described.

### Cytotoxicity tests

#### ARPE-19 and L929 cell cultures

The human retinal pigment epithelial cell line ARPE-19 and the mouse fibroblast L929 cell lines were obtained from the American Tissue Culture Collection (ATCC, VA, USA). After thawing, the ARPE-19 cells were grown in Dulbecco’s Modified Eagle Medium and Ham’s F12 Nutrient Mixture (DMEM/F12, Gibco®, Invitrogen, Paisley, UK), and the L929 cells were grown in DMEM cell culture medium (Gibco®, Invitrogen). Both cell culture media were supplemented with 10% foetal bovine serum (FBS; Gibco®) and 1% antibiotic and antimycotic (100 U/ml penicillin, 100 µg/ml streptomycin, and 0.25 µg/ml amphotericin B; Gibco®). These cell cultures were maintained in 75 cm^2^ flasks (Costar, Corning Inc., Corning, NY, USA) in standard culture conditions of 37 °C and 5% CO_2_, changing the media every 2 days. The cells were harvested by trypsinization using 0.05% Trypsin-EDTA solution (Gibco®) at 80–90% culture confluence and further sub cultivated into culture flasks^8,9^.

#### Preparation of 96-well plates for cell culture

Both ARPE-19 and L929 cells were counted using the Trypan blue (Sigma-Aldrich®, St. Louis, MO, USA) exclusion method quantified by a TC20^TM^ automated cell counter (Bio-Rad Laboratories, Madrid, Spain). The cells were plated in 96 well flat bottom plates using a multichannel pipette. Each 96-well plate was partitioned into columns in the following way: (1) culture media only, i.e., no cells; (2) cells incubated in culture medium alone; (3) positive control cells incubated in culture medium containing a cytotoxic ISO-recommended product, i.e., liquefied phenol meeting USP testing specifications (P9346, Sigma-Aldrich); (4) negative control cells incubated in culture medium with PFO, provided by non-Alamedics manufacturers and commercial companies and not associated with any toxic clinical cases; and (5) test cell cultures incubated in culture media with the suspected toxic PFO samples from Alamedics. All of the conditions were tested in triplicate wells, and each experiment was repeated three times.

The ARPE-19 cells were seeded at a cell density of 10,000 cells per well and grown in 200 µl per well DMEM/F12 culture medium in standard culture conditions for 7 days. Every 2–3 days the culture medium of each culture plate was replaced with fresh culture medium. At 7 days the culture medium was removed, and the culture was incubated for the next 24 hours in culture medium lacking 10% FBS for cell cycle synchronization.

Simultaneously, L929 cells were prepared as single cell suspensions for testing by the extract dilution method as described in different test reports accomplishing ISO standards. At 1.0 × 10^5^ cells per ml of DMEM, 50 µl were plated in each of the 96 wells and incubated under standard culture conditions for the next 24 hours.

#### The effect of culture medium on PFO evaporation

PFO is a volatile liquid, and it is immiscible in water. Therefore we performed a preliminary experiment to establish a protocol for conducting direct exposure tests. An 80 µl PFO sample was placed in two different wells. In one well, 120 µl of culture medium was deposited over the PFO sample. The ratio of PFO sample and culture medium was 40:60 in this study; however, this ratio can be modified in accordance with the needs of the study. After 45 and 90 minutes, the wells were observed to evaluate the PFO layers in each well.

#### Direct contact method with new essential technical steps

The ARPE-19 and L929 cell cultures were washed three times with the corresponding culture media without 10% FBS. For each washing, 200 µl of culture medium was added to each well, and then the plate was inverted with slightly vigorous shaking to discard the culture medium from each well. After the three washes, 80 µl of the PFO samples from non-Alamedics manufacturers and commercial companies and Alamedics (negative controls) and from Alamedics (suspected toxic PFO samples) were directly deposited over cell cultures in each well of corresponding negative group and test group columns. Further, 120 µl of culture medium was deposited over the PFO sample of each well to halt the evaporation of the PFO. The positive control, liquefied phenol (1.6 mg/ml), was prepared in a 3 X concentration in culture medium, and 200 µl was deposited over the cell culture in each well of positive control group column. Similarly, 200 µl of culture medium was deposited over the cultures in each well of the unexposed and blank group columns. The plates were transferred to the incubator regulated at standard conditions for 30 or 60 minutes. After these exposures, the plates were washed as described previously and then incubated for 24 and 72 hours in culture medium (200 µl per well).

At 1 and 3 hours after initiation of the incubation period, each culture was stained with Trypan blue and imaged by phase contrast microscopy (Nikon Eclipse TS100, Nikon España, Barcelona, Spain). These times were selected for early detection of cytotoxicity as manifested by the loss of cell membrane integrity and penetration of the Trypan blue. At the end of each 24- and 72-hour incubation period, the viability of each cell culture was measured using the *3-(4,5-dimethylthiazol-2-Yl)-2,5-diphenyltetrazolium bromide* (MTT) cytotoxicity assay (described below).

#### PFO sample extract preparation

To prepare the PFO for the extract dilution test method, following ISO norms, 0.2 gm of control and test PFO samples were added to 1 ml of DMEM culture medium. For the positive control group, the liquefied phenol (1.6 mg/ml) was prepared in a 3× concentration in 1 ml of DMEM culture medium. Another 1 ml DMEM culture medium was prepared as a control. The mixtures were stirred at 37 °C for 24 hours and used in accordance with the recommendations of the ISO norms. Aliquots of each preparation were collected and prepared in 6 concentrations: 100%, 66.7%, 44.4%, 29.6%, 19.8%, and 13.2% following the calculation 100 × 2/3 = 66.7%, i.e., 40 µl of 100% extract in 60 µl of DMEM culture medium; 66.7 × 2/3 = 44.4%, i.e., 40 µl of 66.7% extract in 60 µl of DMEM culture medium, etc.

#### Extract exposure method

After 24 hours of culture, the L929 culture medium was removed by a slightly vigorous inversion of the plates. Subsequently, each culture well received 200 µl of the control or test samples. The blank wells with no cell cultures and control cultures exposed only to standard culture medium received 200 µl of fresh DMEM. The six different concentrations, 100%, 66.7%, 44.4%, 29.6%, 19.8%, and 13.2% diluted in fresh cell culture medium of the extracts of culture medium, positive and negative controls, and test PFO samples were deposited in the corresponding wells. The cell culture plates were incubated for the next 72 hours in standard culture conditions. After 72 hours, the viability of the L929 cell cultures of each well was measured using the *(sodium 3*′*-[1-(phenylaminocarbonyl)- 3,4-tetrazolium]-bis (4-methoxy-6-nitro) benzene sulfonic acid hydrate)* (XTT) cytotoxicity assay (see below). The XTT assay was selected for this study because it was applied in the reports related to the now presumed toxic PFO samples.

#### Cytotoxicity measurements by the MTT and XTT assays

Upon completion of the incubation periods, the culture medium was removed from the culture plates used for direct exposure testing. Each culture well was washed as described previously. Fresh MTT (Sigma-Aldrich®, St. Louis, MO, USA) solution in DMEM/F12 culture medium without phenol red (Gibco®, Paisley, UK) was prepared in a stock concentration of 5 mg/ml and further diluted in culture medium without phenol red to yield a 10% MTT solution. The MTT solution (50 µl) was deposited in each well, and the cell culture plates were incubated for 2 hours under standard culture conditions. The MTT solution was then removed, and the formed crystals of each well were solubilized by repetitive pipetting using a multichannel pipette containing 100 µl of a solution prepared by dissolving 0.1 N HCL (Panreac Química, Barcelona, Spain) in 2-propanol (Sigma-Aldrich®). After solubilization, the solutions were transferred to fresh flat bottom 96-well plates, and the absorbance (570 nm) of each well was measured by spectrophotometry (SpectraMax® M5, Bionova Cientifica SL, Madrid, Spain). Background absorbance at 650 nm was subtracted from the readings at 570 nm to obtain the final optical density (OD).

The XTT assay was performed as recommended by the manufacturer (Cell Proliferation Kit II (XTT), Roche, Mannheim, Germany). A fresh mixture of XTT labelling reagent (5 ml) and the electron coupling reagent (0.1 ml) was prepared, and 50 µl of this mixture was deposited in each well. The culture plates were transferred to an incubator for 2 hours, and the absorbance of each well was measured at 490 nm (SpectraMax® M5). Background absorbance at 630 nm was subtracted from the readings at 490 nm to obtain the final OD.

#### Data analysis

According to Spanish UNE-EN ISO guidelines (UNE-EN ISO 10993–5 “C.2.3.3 Verification of the Quality of Analysis (II); blank”), two acceptance criteria were included to evaluate the quality of the test analyses. First, the average OD value of group b (in this study, the unexposed group), OD_570_ must be ≥0.2. Second, the average OD value of groups b seeded and grown on both the left and the right sides of the other groups (positive and negative controls and test groups in this study), does not differ more than 15% of the average OD value of the total group b.

The following equation (Spanish Guideline UNE-EN ISO 10993-5 “C.2.5 Data analysis”) was used to calculate the reduction in culture viability of cells exposed to PFO samples in comparison to cell culture viability of group b:

$${\rm{ \% }}{\rm{V}}{\rm{i}}{\rm{a}}{\rm{b}}{\rm{i}}{\rm{l}}{\rm{i}}{\rm{t}}{\rm{y}}={(100\times {\rm{O}}{\rm{D}}}_{570{\rm{e}}}{)/{\rm{O}}{\rm{D}}}_{570{\rm{b}}}$$

where OD_570e_ is the average OD of the respective groups that were in contact with different lots of the product; OD_570b_ is the average OD of all wells of group b. All values are final ODs after subtraction of background absorbance.

A tested product has cytotoxic potential when the cell culture viability decreases to <70% in comparison to the group b, which was set at 100% viability. Finally, the average viability percentages were obtained for each group based on the outcomes of the three repetitions of each experiment.

## Results

### Direct contact method

#### Preliminary study with PFO samples

**Figure 1:**
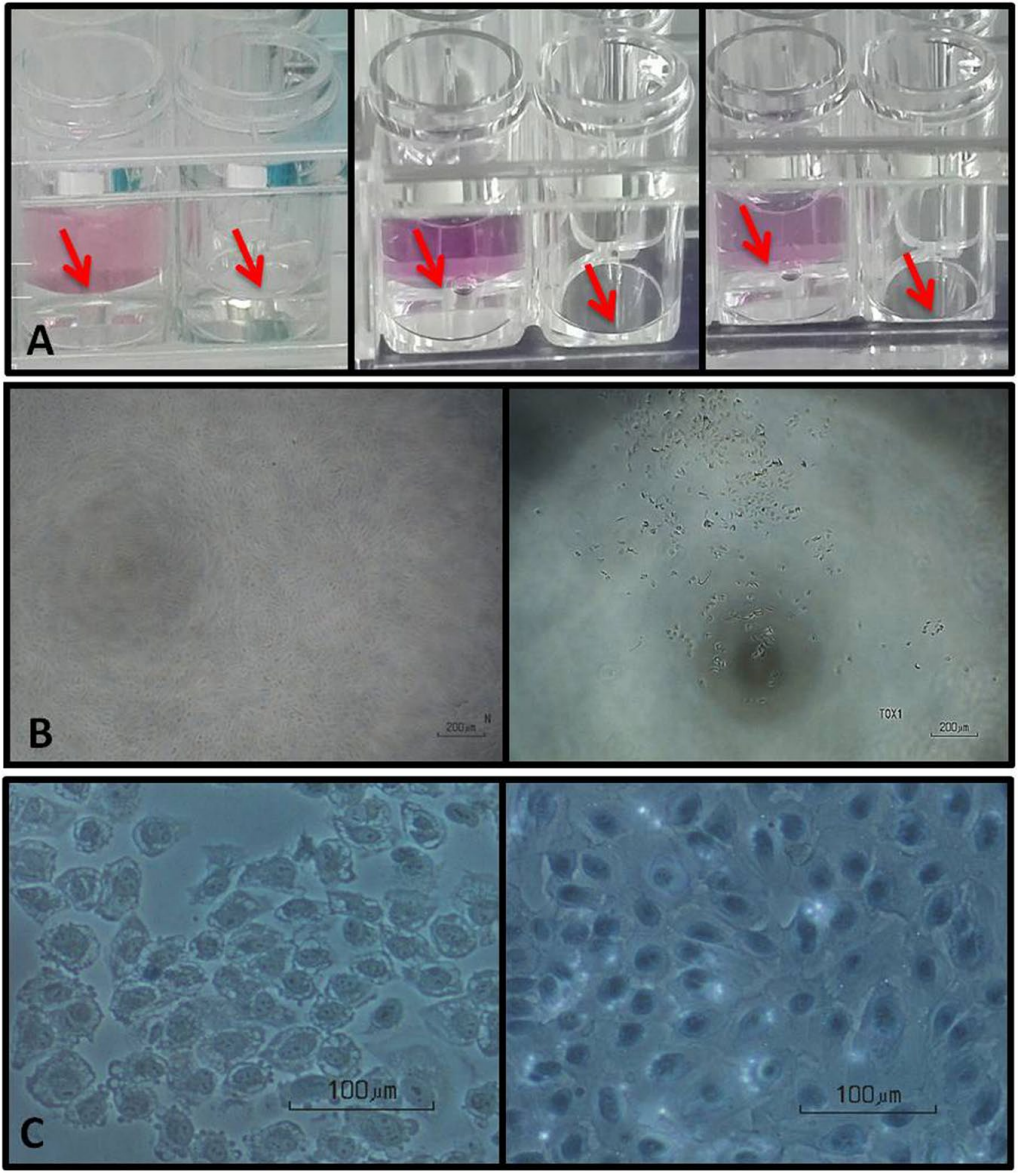
Direct contact method with new essential technical steps to measure cytotoxicity of PFO sample. (**A**) The culture medium (non-volatile liquid) over PFO sample (transparent volatile liquid) retained the quantity of PFO sample in the well by blocking its evaporation. **(A**, left) Initial quantity of PFO and culture medium the wells. **(A**, middle and right) Loss in quantity of PFO sample over time in wells without a layer of culture medium to block evaporation in comparison to the other well (right). Arrow indicates upper surface of PFO sample. (**B**, left) Phase contrast microscopy of ARPE-19 cells incubated for 1 hour after exposure to a control PFO sample. The cells retained confluent organization, and after 3 hours of culture (**C**, left) did not stain with Trypan blue. In contrast, ARPE-19 cells incubated for 1 hour after exposure to a toxic PFO sample (**B**, right) showed a loss of confluency, and after 3 hours of culture (**C**, right) they were heavily stained with Trypan blue. The letter N and word TOX1 in figure 1B are lab internal codings only.

For this study, the ratio between the PFO sample and culture medium was 40:60 (Fig. 1A). The PFO sample did not dissolve in the culture medium, and it was retained at the bottom of the well, below the culture medium. Retention as a separate PFO layer below the culture medium for up to 90 minutes confirmed that it did not evaporate or significantly mix with the overlying culture medium. However, in the absence of an overlying layer of culture medium, the PFO sample layer evaporated (Fig. 1A).

Phase contrast images of cultures taken at 1 hour (Fig. 1B left) and 3 hours after the exposure period showed that the cells in cultures exposed to control PFO samples were confluent and did not stain with Trypan blue (Fig. 1C left). Thus the cells were viable and showed no evidence of being crushed by the 80 µl PFO sample. However, the cell cultures incubated with the suspected toxic PFO sample lost confluency (Fig. 1B right) and stained with Trypan blue (Fig. 1C right).

#### Cell culture quality evaluation for ISO norms

For each experiment, the quality of cell cultures was evaluated and compared to the ISO norms. Viability of the unexposed groups of cell cultures, i.e., cultures exposed only the culture medium, was set at 100% to compare with the responses of the controls and test PFO samples. The cell cultures had a difference <15% between the total mean cell culture viability (left + right columns of the unexposed groups) and the mean cell culture viability (from the left and right column of the unexposed groups). The uniformity in cell culture seeding and growth in the entire cell culture plate was confirmed. In cultures exposed to phenol, the viability was always <70% (data not shown), confirming the cell culture responded appropriately with the positive control. The viability of cultures incubated with PFO samples known not to be toxic and supplied by other manufacturers and companies was always >70%, confirming the cell culture responded appropriately with the negative controls. Cell cultures that did not fulfil these parameters were discarded and not further analysed.

#### Comparison of cell line responses to PFO samples

**Figure 2:**
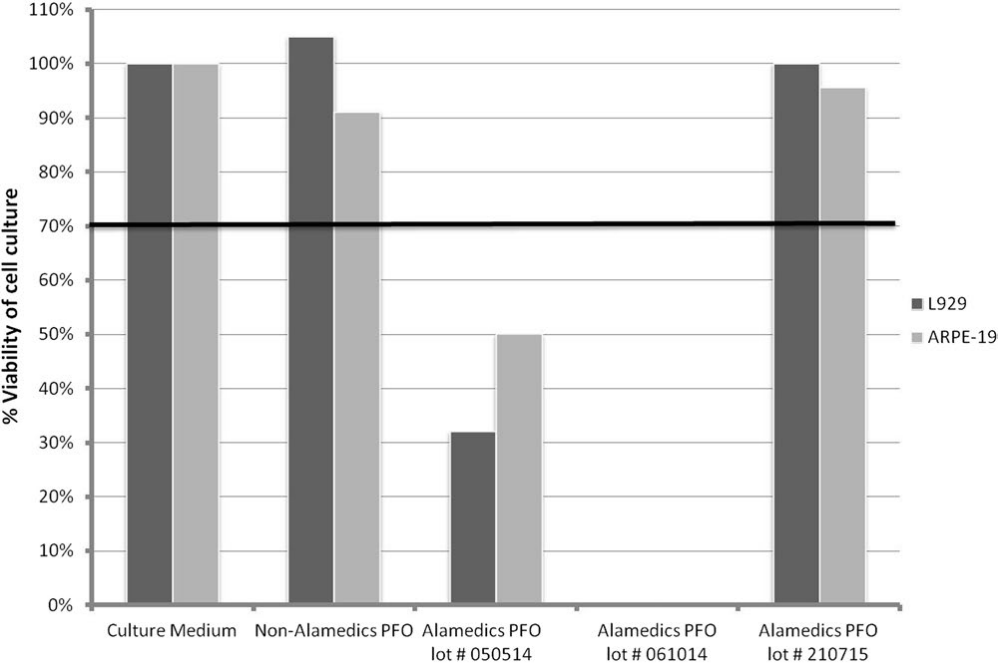
Direct contact method to test viability of L929 and ARPE-19 cultures exposed for 30 minutes to culture medium, non-toxic PFO, and suspected toxic PFO. After exposure, the cultures were grown for 24 hours. Cytotoxicity was determined by the MTT assay. Both cell lines responded similarly to the different samples. Cells exposed to culture medium alone and to culture medium with non-toxic PFO (the non-Alamedics PFO and the Alamedics PFO lot # 210715) had viabilities that exceeded 70%, the cut off for designating the medium as non-toxic or toxic according to ISO norms. Cultures exposed to suspected toxic Alamedics PFO lots # 050514 and # 061014 had viabilities below 70%.

Both the retinal pigment epithelium ARPE-19 and mouse fibroblast L929 cell cultures were directly exposed to control and test samples for 30 minutes and then further grown for 24 hours after washout of the samples. Based on the MTT assay results, the viability of the ARPE-19 and L929 cell cultures compared to a control non-Alamedics PFO sample were 105% and 91.10% respectively (Fig. 2). However, the viability of ARPE-19 cell culture after exposure to the suspected toxic PFO samples from Alamedics lot # PFO 050514 and lot # PFO 061014 was 32% and 0% respectively, both of which were <70%, confirming the toxicity of the PFO lots in accordance with ISO norms. For the L929 cell cultures, the viabilities were 50% and 0.30% for the respective PFO lots, again <70% and confirming the toxic nature of the lots according to ISO norms. The ARPE-19 and L929 cell culture viabilities when exposed to non-suspect PFO samples from Alamedics lot # 210715 were 100% and 95.70%, >70%, confirming the non-toxic properties of the lot in accordance with ISO norms.

#### Comparison of cell culture growth after PFO sample exposure

**Figure 3:**
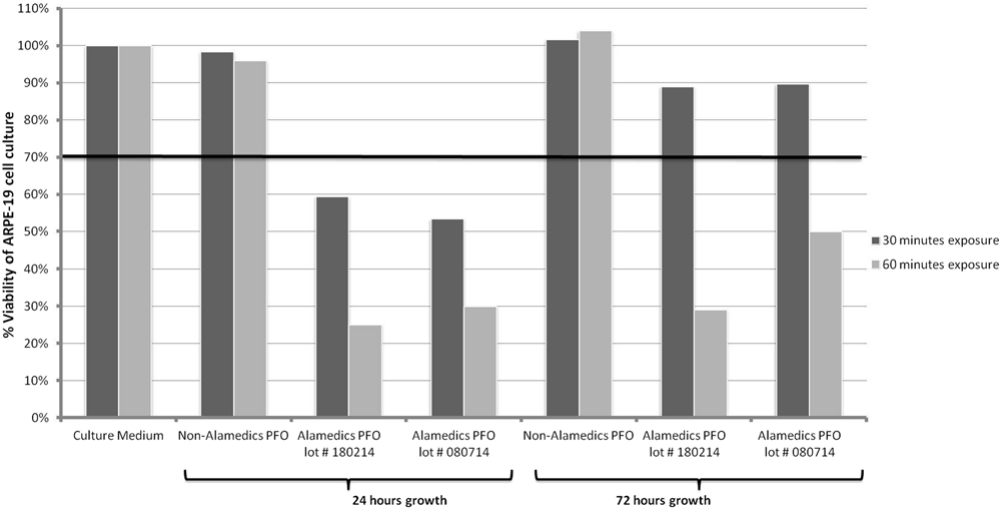
Comparison of ARPE-19 cell culture viability at 24 and 72 hours after 30 and 60 minutes of PFO exposure. After 30 minutes of exposure to Alamedics PFO lots # 180214 and # 080714, the viabilities were <70% and considered to be toxic at 24 hours of culture. However at 72 hours, the viabilities were >70% and not considered to be toxic. After 60 minutes of suspected toxic PFO exposure, cell viabilities at both 24 and 72 hours were <70%. Hence selection of parameters such as exposure times and post-exposure growth periods are important for applying the direct contact method.

The ARPE-19 cell cultures were directly exposed to samples for 30 and 60 minutes and then grown for 24 and 72 hours. The MTT assay results at 24 and 72 hours incubation after 30 minutes exposure to non-Alamedics PFO showed that the viabilities were 98% and 102% of the non-exposed cultures that were grown in culture medium alone (Fig. 3). However, the viabilities of cell cultures exposed to the suspected toxic Alamedics PFO lot # 180214 for 30 min were 59% and 89% after 24 and 72 hours growth in culture medium. In cultures exposed for 30 minutes to the suspected toxic Alamedics lot # 080714, the viabilities were 53% and 90% at 24 and 72 hours of continued growth in culture. These results demonstrated that tests of Alamedics PFO lots 180214 and 080714 had cell culture viabilities <70% at 24 hours and were toxic based on ISO standards. However at 72 hours, the viability of these cultures exceeded 70% and therefore the PFO could be considered non-toxic by the ISO standards.

The results for 60 minutes exposure to non-Alamedics PFO followed by 24 and 72 hours growth showed that ARPE19 culture viability compared to the cultures not exposed to PFO was 96% and 104% respectively. However, cultures exposed to Alamedics PFO lot # 180214 had only 25% and 29% viability at 24 and 72 hours. Cultures exposed to Alamedics PFO lot # 080714 had 30% and 50% viability at 24 and 72 hours respectively. These results demonstrated that Alamedics PFO lots suspected of having cellular toxicity were indeed so, with viabilities <70% when the exposure period was extended to 60 minutes. The non-Alamedics control PFO lot was always non-toxic as confirmed by cell culture viabilities >70% at 24 and 72 hours of post-exposure growth periods.

#### Extract dilution exposure method

**Figure 4:**
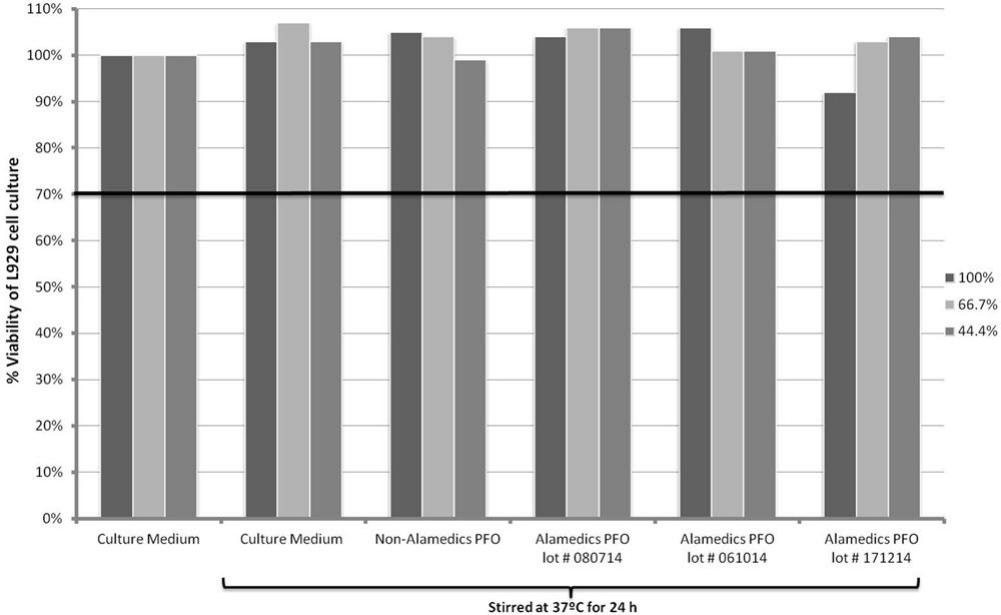
Viability of L929 cell cultures exposed to different PFO extract concentrations. The cultures were exposed to PFO at 100%, 67.7%, and 44.4% and then cultured for 72 hours. Viability was measured by the XTT assay. This extract dilution exposure protocol did not detect toxicity in any of the PFO samples.

The XTT assay results (Fig. 4) at 72-hour growth periods showed that the L929 cell culture viability with PFO extracts concentrations of 100%, 66.7% and 44.4% of culture medium (prepared using extract method) were 103%, 107% and 103% respectively. For the control non-Alamedics PFO sample at 100%, 66.7%, and 44.4% strengths, the viabilities of the cultures at 72 hours of culture were 105%, 104% and 99% respectively. For the suspected toxic Alamedics PFO lot # 080714, the viability at 100% strength was 104%, and at 67.7% and 44.4% strengths the viabilities were 106% and 106% respectively. For the suspected toxic Alamedics PFO lot # 061014, the viability at 100% strength was 106%, and at 67.7% and 44.4% strengths the viabilities were 101% and 101% respectively. Finally, for the suspected non-toxic Alamedics PFO lot # 171214, the viability at 100% strength was 93%, and at 67.7% and 44.4% strengths the viabilities were 103% and 104% respectively. These results showed that exposure of L929 cells to any of the PFO samples extracts did not negatively affect the viability after 72 hours of growth. Thus, these data suggest that the suspected toxic lots of Alamedics PFO were not toxic to the cultured L929 cells under the conditions of this assay. Because of the high viability of cultured fibroblasts exposed to the extracts of PFO at dilutions of 100%, 66.7%, and 44.4%, we did not evaluate viabilities of L929 cultures exposed to even more dilute PFO extracts.

#### Summary of comparison between direct contact and extract exposure method

The Alamedics suspected toxic PFO sample lots # 180214 and #150414 were taken from Container 1 (Table 1). The viabilities of the human retinal pigment epithelial cell line ARPE-19 for these lots, determined by the direct contact method after a 30-minute exposure followed by 24 hours of culture, were 59% and 34% respectively. The viability of cells exposed to Alamedics PFO # 180214 for 60 min followed by 24 hours of culture was 25%. Container 2 was the source of Alamedics PFO lots # 061014 and # 171214, and exposure of ARPE-19 cells to either lot resulted in only 1% viability. PFO lot # 080714 was prepared by mixing PFO samples from Alamedics Containers 1 and 2, and the viabilities after exposure for 30 or 60 minutes were 53% and 30% respectively. However, mouse fibroblast L929 cell cultures had viabilities >70% at 72 hours of incubation after exposure to all of the PFO lots. As in the direct exposure method, the negative control PFO lots from non-Alamedics manufacturers {and all of the Alamedics PFO lots} showed >70% cell culture viability with the extract dilution exposure method in which the various dilutions were prepared after stirring the samples for 24 hours at 37 °C. PFO samples of many other manufacturers and companies, and many other test lots were also analysed for validation of the extract exposure protocol, and similar results were obtained (data not shown).

## Discussion

Cytotoxicity testing is a primary and crucial step that can decide the fate of a health-related product for clinical application. The ISO guidelines recommend standard parameters for performing cytotoxicity tests. Recently several cases of blindness after non–complicated retinal detachment surgery in which PFO was used have been reported in Spain, Chile, and other countries^5^. This indicates that cytotoxicity tests applied by some manufacturers for the clinically applied PFO lots are inadequate. Investigation of these cases showed that most were attributed to toxic lots of PFO used during the surgeries. The methods applied for certifying PFO, which are performed before clinical use, were unable to detect the toxicity. Typically, the test protocols used to detect cellular toxicity were the extract dilution method and the indirect contact method in which the PFO samples are not in contact with cells. Furthermore, the tests utilized the L929 cell line, a popular source of mouse fibroblasts, but which is reported to be more resistant to the toxicity of certain test agents^10^.

An important consideration for selecting resources used during cytotoxicity testing is the cells and tissues that are likely to be affected by the tested agent. PFO used in the ophthalmology clinical setting is in contact with human retina and associated cell layers, including the retinal pigment epithelium. In this study, we proposed a crucial new technical protocol based on the direct contact method with cell cultures of the ARPE-19 cell line that is derived from human retinal pigment epithelium (RPE).

The ARPE-19 cell line is readily available, widely used in investigations, and is easily grown and maintained under laboratory conditions. The cultures of other retinal cells, such as ganglion cells or cells found close to the vitreous, have not been properly established, and primary cultures of retinal cells are difficult to grow and maintain. The L929 cells of mouse fibroblast origins are also well established and easily grown in the lab, but, while they have been used in other studies, including the ones testing PFO toxicity, they may not adequately reflect the response of retinal and related tissues to PFO. Thus, ocular toxicity studies would be more convincing if the tests were performed on cell types that are of mammalian retinal origin. The ARPE-19 cells meet the requirements of availability, reliable growth under standard laboratory conditions, and of mammalian retinal origin.

PFO liquid is a volatile, high molecular weight polymer^11^. It has been considered that the heaviness of a PFO sample could crush the cell culture, and that the volatile nature could make it difficult to maintain the PFO concentration during the cell culture exposure period. To examine these important issues, we added 80 µl of PFO samples to the ARPE-19 and L929 cultures. We then overlaid the PFO with 120 µl of culture medium. Selection of the 80 µl and 120 µl volumes depended on ease of visibility of the two layers. Additionally, the 40:60 ratios for the PFO sample and culture medium was considered adequate considering the volumes of PFO used in clinical practice. However, other proportions can be used for performing such *in vitro* studies. We found that the culture medium effectively blocked PFO evaporation. This confirmed that this method provided an important step that retains the volatile substance without any apparent loss due to evaporation or dilution by mixing with the culture medium. Further, phase contrast microscopy of cultured cells exposed to the non-toxic PFO did not suggest that the cells were damaged due to the weight of the PFO sample. They retained a confluent appearance and were not stained with Trypan blue, confirming cell viability. The high molecular weight, density and hydrophobic properties contributed to retain the PFO sample layer without floatation and below culture medium layer directly in contact with cells of cell culture. However, in cell cultures incubated with suspected toxic PFOs, the confluent appearance was lost, and the cells stained with Trypan blue, indicating the loss of cellular integrity.

The L929 cell culture, composed of mouse fibroblasts, is used for determining PFO cytotoxicity by the extract dilution exposure method. However, because PFO is applied during retinal detachment surgery, it is more logical to conduct preliminary tests of toxicity in cultures of retinal cells. In fact, the UNE-ISO standards state that, as far as possible, the testing procedures should be similar to the clinical situation in which the product will be used. The use of L929 cell culture for testing PFOs to be used in retinal surgery clearly does not meet this requirement. In contrast, the ARPE-19 cell line, derived from human retinal pigment epithelial cells, is very popular and readily available for research purposes, including toxicity testing. We prepared both types of cultures and exposed them to different PFO samples for 30 minutes, and then analysed them at 24 hours by the MTT assay to determine the suitability of each to detect PFO cytotoxicity. Control groups of both cell lines exposed to culture medium alone or to non-toxic PFO sample from other manufacturers and companies and from non-toxic Alamedics PFO lot # 210715 had viabilities >70% and typically they were around 100%. However cultures of both cell lines exposed to suspected toxic Alamedics PFO sample lots # 050514 and #061014 had viabilities <70%, typically between 0% and 50%. These data confirmed that both cell cultures types responded differently to non-toxic and toxic PFO lots. Thus, the failure to identify the toxicity of AlaOcta® before clinical use can be attributed to the inappropriate selection of the testing method, i.e., extract dilution, in which the cell type, i.e., L929 was used.

It is logical that PFO samples from Alamedics lot #061014, which produced 100% mortality in both ARPE-19 and L929 cell cultures at 24 hours, should have no growth at 72 hours. However cultures of ARPE-19 cell exposed for 30 minutes to Alamedics lot #050514, which was considered to be toxic, had >70% viability at 72 hours of post-exposure growth. This could be attributed to the fact that the lot induced only 40–50% mortality at 24 hours after PFO exposure. The surviving cells at 24 hours likely exhibited sufficient growth over the next 48 hours to achieve a viability >70%. Due to the limited amount of toxic PFO sample per lot, the experiments and results were restricted to only ARPE-19 cell cultures. Nevertheless, our results clearly showed that the L929 cell cultures were not resistant to PFO toxicity when it was applied appropriately for a volatile substance.

The protocol steps of any test method are very crucial for detecting mild to strong cytotoxicity. For this purpose, ARPE-19 cell cultures were exposed to PFO samples and further grown for the next 24 and 72 hours and analysed by the MTT cytotoxicity assay. All of the cultures exposed to the culture medium alone or to control non-toxic PFO samples had viabilities at or near 100% at 24 and 72 hours. In contrast, cell cultures exposed to suspected toxic PFO lots # 180214 and # 080714 for 30 minutes had viabilities <70% at 24 hours. However by 72 hours of post-exposure growth, the viabilities exceeded 70%. Thus, this test method detected the toxic nature of the PFO samples only at 24 hours. By 72 hours of post-exposure growth, the viability had recovered to >70%. However, the results for 60 minutes exposure followed by 24 and 72 hours growth periods showed that the ARPE-19 cell culture viability was 25% and 29% for lot # 180214 and 30% and 50% for lot # 080714 respectively. Thus, after 60 minutes of exposure, the toxic nature of PFO lots # 180214 and # 080714 was evident at both culture times. Hence, these results confirmed that extending the exposure period from 30 to 60 minutes is a crucial step for detecting toxic PFO samples at 72 hours of post-exposure growth. These findings provide a crucial step in the testing protocol. In general, we recommend measuring the cell culture viability at more than one timepoint.

In further studies, extract dilutions of suspected toxic Alamedics PFO sample lots # 080714, # 061014, and # 171214 were incubated with L929 cell cultures for 72 hours, followed by XTT assay for measuring cell culture cytotoxicity. All of the cell cultures retained viability >70%. The extract dilution method is based on the idea that leachable toxic components of the toxic PFO samples would be released and miscible with the culture medium^12^. This does not seem to have occurred in this case. If they were released, the amount was insufficient to induce any detectable cellular toxicity. These results highlight a major advantage of the direct contact method over the extract dilution method. The direct contact method ensures that the cells are exposed directly to the agent being tested. It is more likely to provide meaningful data regarding the potential toxicity of PFO or any other compound under study.

The L929 cells were used in the current procedures as recommended by ISO guidelines. However performing a test over retinal cell cultures is more realistic because the PFO product in question is used in retinal surgery. Toxicity testing of a toxic PFO sample of another manufacturing company was based on the extract exposure method as applied to ARPE-19 cells. The study failed to detect toxicity, as reported previously with L929 cell cultures. Here, however, we have successfully detected the toxicity by applying our new direct contact method to ARPE-19 cells^13^.

We applied both the direct contact and the extract dilution methods with many test PFO samples of different manufacturers and companies using ARPE-19 and L929 cell cultures to reach conclusions and propose a better, more reliable method of testing. Table 1 presents a comparison of the methods and the outcomes. The results obtained by the direct contact method, performed in accordance with our proposed new technical protocol, clearly demonstrate that the Alamedics PFO samples that we tested became more cytotoxic over time. For instance, using our direct method of testing with ARPE-19 cells, in Frebruary 2014 the viability of Alamedics lots was 50% and clearly toxic. By December 2014, the viability was only 1%. However, the extract dilution method was unable to detect this cytotoxicity. Thus, these results confirmed that the proposed new direct contact method is better than the extract dilution method for detecting cytotoxicity of health-related products such as PFO.
